# ARID1B as a Potential Therapeutic Target for ARID1A-Mutant Ovarian Clear Cell Carcinoma

**DOI:** 10.3390/ijms19061710

**Published:** 2018-06-08

**Authors:** Emi Sato, Kentaro Nakayama, Sultana Razia, Kohei Nakamura, Masako Ishikawa, Toshiko Minamoto, Tomoka Ishibashi, Hitomi Yamashita, Kouji Iida, Satoru Kyo

**Affiliations:** Department of Obstetrics and Gynecology, School of Medicine, Shimane University, Enyacho 89-1, Izumo, Shimane 6938501, Japan; prettynanaone@gmail.com (E.S.); raeedahmed@yahoo.com (S.R.); kohei320@med.shimane-u.ac.jp (K.N.); m-ishi@med.shimane-u.ac.jp (M.I.); minamoto@med.shimane-u.ac.jp (T.M.); tomoka@med.shimane-u.ac.jp (T.I.); memedasudasu1103@gmail.com (H.Y.); iida@med.shimane-u.ac.jp (K.I.); satoruky@med.shimane-u.ac.jp (S.K.)

**Keywords:** ARID1A, ARID1B, ovarian clear cell carcinomas, progression-free survival

## Abstract

AT-rich interactive domain 1A (ARID1A) and AT-rich interactive domain 1B (ARID1B) are subunits of the SWI/SNF chromatin complex. ARID1A is a tumor suppressor gene that is frequently mutated (46%) in ovarian clear cell carcinomas (OCCC). Loss of ARID1B in an ARID1A-deficient background eliminates the intact SWI/SNF complex, indicating that ARID1B is essential for the formation or stabilization of an intact SWI/SNF complex and, thus, the survival of ARID1A-mutant cancer cell lines. In this study, we investigated the clinicopathologic and prognostic relevance of ARID1B in OCCC by immunohistochemical analysis of 53 OCCC patient samples and loss-of-function experiments in OCCC cell lines. We also examined whether ARID1B could be a therapeutic target or prognostic biomarker in OCCC. siRNA-mediated knockdown of ARID1B in an ARID1A-mutant cell line significantly decreased cell growth, whereas concurrent depletion of both ARID1A and ARID1B was required to decrease wild type cell growth. In the immunohistochemical analyses, low ARID1B level was frequent in samples lacking ARID1A and was associated with shorter progression-free survival. This is the first report demonstrating that a low ARID1B level could be a marker of poor prognosis in OCCC. Moreover, the correlation between the loss of ARID1A immunoreactivity and reduced ARID1B levels indicates that ARID1B could be an attractive target for anti-cancer therapy.

## 1. Introduction

Ovarian cancer is the most lethal gynecological malignancy in the world [1] and its incidence has increased in the last decade. Of the several subtypes of epithelial ovarian cancer, ovarian clear cell carcinoma (OCCC) constitutes nearly 25% of cases in Japan and is genetically distinct from the other histological types, with a worse prognosis due to a lower response rate to anti-cancer drug treatment [2,3,4]. *AT-rich interactive domain 1A* (*ARID1A*) is a novel tumor suppressor gene that is frequently mutated in OCCC; *ARID1A* mutations were identified in 55/119 (46%) OCCC patients in one study with a strong correlation between OCCC and loss of BAF250a protein and the presence of *ARID1A* mutations [5].

ARID1A is a Switch/Sucrose non-fermentable (SWI/SNF) chromatin remodeling complex subunit that regulates many cellular processes such as development, proliferation, differentiation, DNA repair, and tumor suppression [6]. Mutation or loss of *ARID1A* can affect the expression or stability of other SWI/SNF complex subunits. It was recently reported that inactivating mutations in *ARID1A* create a dependency on ARID1B [7]. ARID1A and ARID1B demonstrate nearly 80% amino acid sequence homology in the ARID domains and around 50% homology overall [8]. However, despite the similarity in their amino acid sequences, they have distinct expression patterns [9] and functions (anti- and pro-proliferative for ARID1A and ARID1B, respectively) [10] in development and cell cycle control. In addition, *ARID1A* and *ARID1B* alleles are frequently co-mutated in cancer, but *ARID1A*-deficient cancer retains at least one functional *ARID1B* allele [7]. This suggests that the presence of *ARID1B* is essential for stabilizing the SWI/SNF complex in *ARID1A*-mutant cancer cells.

In this study, we investigated the clinicopathologic and prognostic roles of ARID1B in OCCC by immunohistochemical analysis of ARID1B expression in 53 OCCC patient samples and loss-of-functions experiments in OCCC cell lines. We also examined whether ARID1B can serve as a therapeutic target or a prognostic biomarker in OCCC.

## 2. Results

### 2.1. Effects of ARID1A and ARID1B Knockdown on OCCC In Vitro

**Figure 1:**
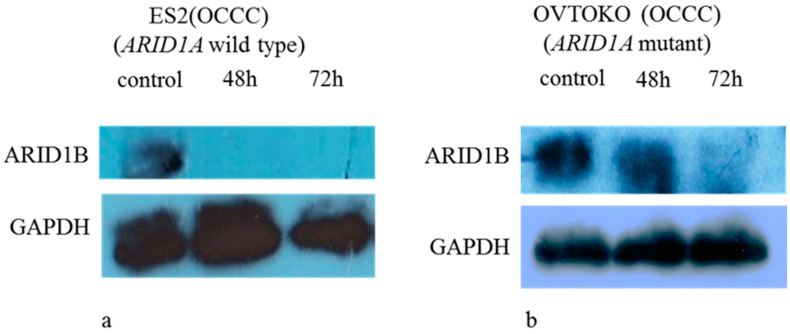
Western blot analysis of ARID1B expression in ovarian clear cell carcinomas (OCCC) cells. ARID1B protein level was decreased after siRNA-mediated *ARID1B* knockdown in wild-type (**a**) and *ARID1A*-mutant (**b**) cells.

**Figure 2:**
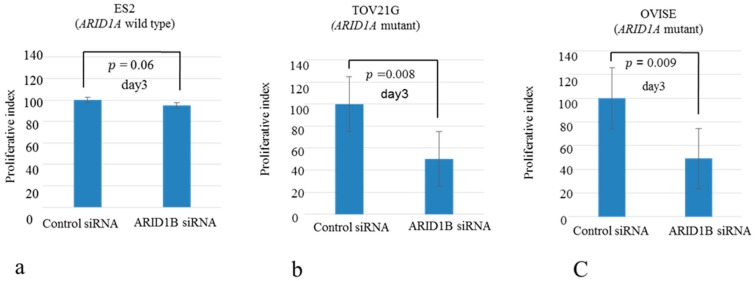
Effects of *ARID1B* knockdown on cell proliferation in OCCC cell lines. (**a**) Wild-type cells were unaffected by *ARID1B* knockdown; (**b**,**c**) *ARID1B* knockdown inhibited proliferation in all *ARID1A*-mutant cells lines.

**Figure 3:**
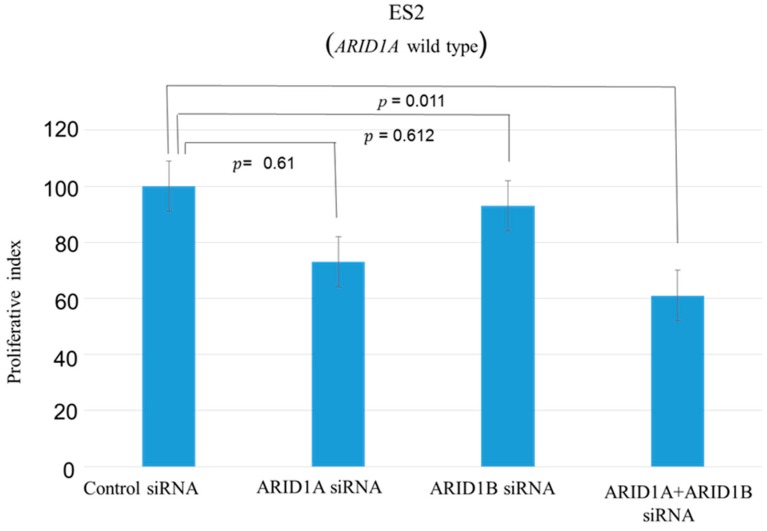
Effects of *ARID1A*, *ARID1B*, *ARID1A*, and *ARID1B* knockdown in OCCC cells without *ARID1A* mutation (wild type). Cell proliferation was inhibited only by concurrent knockdown of *ARID1A* and *ARID1B* and not by knockdown of individual genes.

A panel of ovarian clear cell lines that harbored *ARID1A* mutations was used in this study [11]. SiRNA-mediated knockdown of *ARID1B* in *ARID1A*-mutant (OVISE, OVMANA, OVTOKO, OV207, and TOV-21G) and wild-type (ES2) cell lines reduced ARID1B protein level (Figure 1, supplementary Figures S1 and S2), which was associated with reduced cell growth in *ARID1A*-mutant but not the wild-type cell line (Figure 2, supplementary Figure S3). The proliferation of ES2 cells was inhibited only by concurrent depletion of both *ARID1A* and *ARID1B* (Figure 3).

### 2.2. Relationship between ARID1A and ARID1B Protein Expression in OCCC

**Figure 4:**
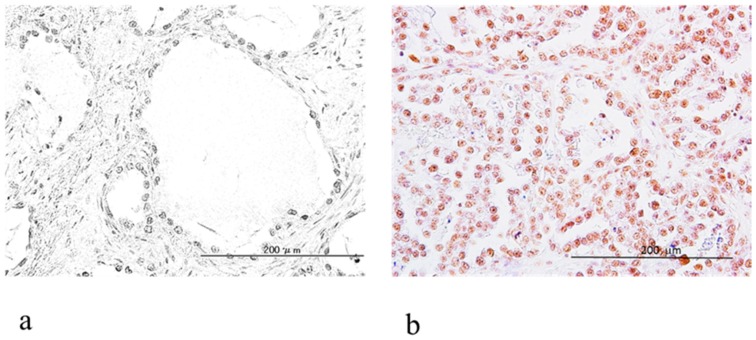
ARID1B expression in clinical OCCC specimens. (**a**) A case of low ARID1B immunoreactivity (upper right panel); (**b**) Intense ARID1B immunoreactivity was observed in the nuclei of OCCC cells (upper left panel).

We analyzed ARID1B expression in 53 OCCC samples; ARID1A expression level in these OCCC samples have been previously described [12]. Eight of the samples were omitted from this study because of the loss of material. From data collected in our earlier study [12], we identified 7/53 (13.2%) OCCC samples with loss of ARID1A expression (Table 1). ARID1B immunoreactivity was detected in tumor cell nuclei (Figure 4a,b) and high expression was observed in 45/53 (85%) samples whereas low expression was observed in 8/53 (15%) samples. Low levels of ARID1B (score < 100) were more frequently observed in samples lacking ARID1A expression (*p* < 0.001, Fisher’s test) (Table 1).

### 2.3. Relationship between ARID1B Expression and Clinicopathologic Factors

We examined the clinical significance of low and high ARID1B expression in OCCC. There was no significant association between ARID1B expression level and FIGO stage, cancer antigen (CA)125 level, patient age, endometriosis, or residual tumor status (Table 2).

### 2.4. Relationship between ARID1B Protein Expression and Progression-Free Survival

**Figure 5:**
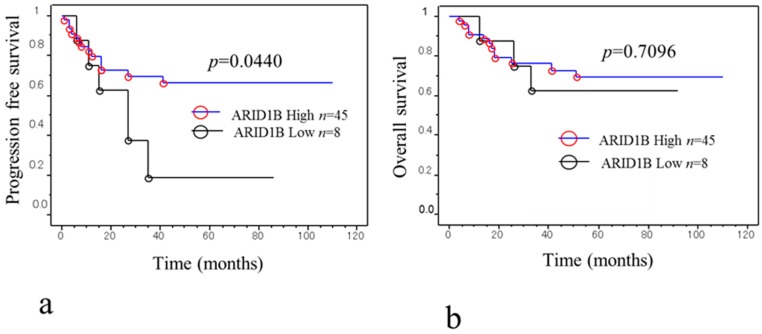
Kaplan-Meier survival analysis of 53 OCCC patients. (**a**) Low ARID1B level (black line, *n* = 8) in OCCC patients is associated with shorter progression-free survival as compared to high expression (blue line, *n* = 45) (*p* = 0.044, log-rank test); (**b**) Low (black line, *n* = 8) and high (blue line, *n* = 45) ARID1B expression had no influence on the overall survival of OCCC patients (*p* = 0.7096, log-rank test).

We investigated whether ARID1B expression level is related to patient outcome by Kaplan-Meier analysis of progression-free survival (Figure 5a). Of the 53 patients, eight with low ARID1B expression had shorter progression-free survival than those with high expression (*p* = 0.044, log-rank test) (Figure 5a). A univariate analysis showed that FIGO stage III/IV (*p* < 0.0001, log-rank test), CA125 level (*p* = 0.0088, log-rank test), residual tumor diameter ≥2 cm (*p* < 0.0001, log-rank test), and low ARID1B expression (*p* = 0.04, log-rank test) were correlated with shorter progression-free survival (Table 3). When the data were stratified in the multivariate analysis, only CA125 level (*p* = 0.0446) and low ARID1B expression were significantly associated with shorter disease-free survival (*p* = 0.03) for (Table 4).

### 2.5. Relationship between ARID1B Protein Expression and Overall Survival

We examined the prognostic significance of low and high ARID1B expression on overall survival but found no correlation between ARID1B level and overall survival (Figure 5b).

## 3. Discussion

The incidence of OCCC is increasing at a higher rate in Japan than in Europe or the United States [13]. OCCC has a poor prognosis due to greater resistance to chemotherapy than other types of ovarian cancer. Therefore, identifying new therapeutic targets in OCCC is especially critical for the Japanese population. *ARID1A* is one of the most frequently mutated genes in many cancer types, particularly ovarian, breast, gastric, and lung cancers [11]. Mutation of ARID1B is rarer than that of ARID1A in human cancer, but has been found in hepatocellular carcinoma and neuroblastoma [14,15]. Sausen et al. [14] and Fujimoto et al. [15] performed whole-genome sequencing analysis and found that neuroblastoma tumors and hepatocellular carcinoma were driven by recurrent mutations in genes including *ARID1A* and *ARID1B* involved in chromatin remodeling. ARID1A and ARID1B are typically co-expressed in cancer, but cancer driven by *ARID1A* mutation retains at least one functional *ARID1B* allele [7]. Establishing the clinical relevance of *ARID1A* mutation and the role of ARID1B can lead to the development of more effective treatments for ovarian cancer.

We addressed this in the present study using five OCCC cell lines (OVISE, OVMANA, OVTOKO, OV207, and TOV-21G) harboring *ARID1A* mutations. SiRNA-mediated knockdown of *ARID1B* suppressed cell growth in *ARID1A*-mutant but not the wild-type cell line (Figure 2, supplementary Figure S3); in the latter, proliferation was inhibited only by silencing both *ARID1A* and *ARID1B* (Figure 3). These results provide evidence for the functional redundancy of *ARID1A* and *ARID1B*.

A previous study reported that *ARID1B* knockdown suppressed growth and colony formation of *ARID1A*-deficient ovarian cancer cell lines [7]. Concurrent *ARID1A* and *ARID1B* mutations were detected in the undifferentiated components of approximately one-quarter of dedifferentiated endometrial and ovarian carcinomas [16]. ARID1A and ARID1B are the only known DNA-binding proteins in the SWI/SNF-A complex; inactivation of ARID1B in an *ARID1A*-deficient background can disrupt SWI/SNF complex activity, thereby contributing to the development and progression of human cancers.

We also examined the relationship between ARID1A and ARID1B protein expression in 53 OCCC patient specimens by immunohistochemistry. ARID1A immunoreactivity was detected in tumor cell nuclei, as we previously reported [12]. ARID1A nuclear expression was observed in 13.4% of samples (Table 1) whereas ARID1B was expressed in all samples at high (85%) or low (15%) levels (Figure 4a,b). Samples lacking ARID1A expression in the nucleus were more likely to exhibit low ARID1B immunoreactivity (Table 1), which was also associated with shorter progression-free survival (Figure 5a). On the other hand, overall survival was unaffected by ARID1B expression level (Figure 5b). The multivariate analysis showed that only CA125 level (*p* = 0.0446) and low ARID1B expression were significantly associated with progression-free survival (Table 4). Our results indicate that inactivating mutations in ARID1A are associated with lower expression of ARID1B. The reason for the association between lower expression of ARID1B and loss of ARID1A is not yet understood. Our previous report found that ARID1A loss was significantly correlated with shorter progression-free survival in patients with OCCC treated with platinum-based chemotherapy [12]. Loss of ARID1A might be correlated with lower expression of ARID1B, which may create a specific vulnerability in ARID1B compared to cells without ARID1A mutations. These results suggest that low ARID1B expression can potentially serve as a prognostic biomarker in patients with recurrent OCCC. However, additional studies with a larger study population are required to establish the percentage of OCCC cases with low ARID1B expression and to determine the association between loss of *ARID1A* immunoreactivity and downregulation of ARID1B. Further investigation is necessary to determine the molecular mechanism underlying ARID1A mutation and ARID1B downregulation. In addition, real time PCR and in vitro chemosensitivity testing are required for evaluation of the expression of ARID1A/ARID1B.

## 4. Materials and Methods

### 4.1. Tissue Samples

Formalin-fixed, paraffin-embedded tissue samples of 53 OCCC patients were used in this study. Samples were obtained from the Department of Obstetrics and Gynecology at the Shimane University Hospital and Seirei Hamamatsu General Hospital. Diagnoses were made based on conventional histopathologic examination of sections stained with hematoxylin and eosin (H&E), and tumors were classified according to World Health Organization criteria. Tumors were staged according to the International Federation of Gynecology and Obstetrics (FIGO) classification. Cytoreductive surgery, along with adjuvant platinum and taxane or CPT-11 chemotherapy, is the primary mode of treatment for patients (carboplatin AUC5 with 175 mg/m^2^ paclitaxel or 70 mg/m^2^ docetaxel or 60 mg/m^2^ CDDP with 180 mg/m^2^ CPT-11). Six-12 cycles of this combination regimen was administered by all patients. All patients were administered adjuvant chemotherapy. Acquisition of tissue specimens and clinical information was approved by the institutional review boards of Shimane University and Serrei Hamamatsu General Hospital. Paraffin tissue blocks were organized into tissue microarrays made by removing 3-mm diameter cores of tumor tissue from the block. The cored area was selected by a gynecological oncologist (KN) and pathology technician (KI) based on review of H&E-stained sections.

### 4.2. Immunohistochemistry

ARID1B expression was evaluated by immunohistochemistry using a mouse monoclonal antibody against ARID1B (BAF250b) (Abcam, Cambridge, MA, USA; ab54761). Immunohistochemical detection of ARID1B was performed on the tissue microarrays and 3 of each core were included at 1:800 dilution followed by the peroxidase method using the EnVision + system (Dako, Carpinteria, CA, USA). After antigen retrieval in sodium citrate buffer, slides were incubated overnight at 4 °C with antibody and then examined under a light microscope by two researchers who were blinded to the clinicopathologic factors. ARID1B immunoreactivity was scored by the two investigators as follows: density, 0 = undetectable, 1 ± weakly positive, 2 ± moderately positive, and 3 ± intensely positive; and range, 0–100%. The two scores were multiplied and those <100 were considered low, whereas those ≥100 were considered high.

### 4.3. Cell Lines and Culture

OVISE, OVMANA, and OVTOKO OCCC cell lines were obtained from the Japanese Health Science Research Resources Bank (Osaka, Japan). The OV207 OCCC cell line was a gift from Vijayalakshmi Shridhar (Mayo Clinic, Rochester, MN, USA). ES2 and TOV-21G CCC human ovarian cancer cell lines were obtained from the American Type Culture Collection (Rockville, MD, USA).

### 4.4. Silencing of ARID1A and ARID1B Gene Expression

Short interfering (si) RNAs against *ARID1A* and *ARID1B* were purchased from Santa Cruz Biotechnology (Santa Cruz, CA, USA). Cells were seeded in 6-well plates and transfected with siRNA using Oligofectamine reagent (Invitrogen, Waltham, MA, USA), and collected 48 h later for western blot analysis of ARID1A and ARID1B protein levels.

### 4.5. Western Blot Analysis

OVISE, OVMANA, OVTOKO, OV207, TOV-21G, and ES2 cells were used for western blotting. The cell pellets were dissolved in Laemmli sample buffer (Sigma-Aldrich, St. Louis, MO, USA) to obtain the cell lysates. From each lysate, the total proteins were loaded (equal amount) and separated using gels containing 10% Tris-glycine sodium dodecyl sulfate polyacrylamide (Novex, San Diego, CA, USA), and the bands were electrotransferred to an Immobilon-P polyvinylidene difluoride membrane (Millipore, Billerica, MA, USA) that was probed with anti-ARID1B antibody (1:1000; Santa Cruz Biotechnology) followed by peroxidase conjugated anti- rabbit immunoglobulin (1:20,000; Santa Cruz Biotechnology, Dallas, TX, USA).The same membrane was then probed using an antibody that reacted with glyceraldehyde 3-phosphate dehydrogenase (1:10,000; Cell Signaling Technology, Danvers, MA, USA) as a loading control. Protein bands were visualized using a chemiluminescence kit (Pierce, Rockford, IL, USA).

### 4.6. Cell Proliferation Assay

Cells were seeded in 96-well plates at a density of 3000 cells/well. Cell number was indirectly determined with the 3-(4,5-dimethylthiazol-2-yl)-2,5-diphenyltetrazolium bromide) tetrazolium (MTT) reduction assay [17] 96 h after treating the cells with siRNAs against ARID1A, ARID1B, or ARID1A + ARID1B or control siRNA. Data are expressed as mean ± standard deviation of the dimethylsulfoxide control from triplicate determinations in three independent experiments.

### 4.7. Statistical Analysis

The *t* test was used to evaluate the statistical significance of the results of the MTT assay using SPSS statistical software (Version 19, SPSS Inc., Chicago, IL, USA). *p* values <0.05 were considered statistically significant. Fisher’s exact test was used for comparisons of categorical data. Overall survival was calculated as the time between disease diagnosis and death or last follow-up. Kaplan-Meier curves were used to plot the survival data and log-rank tests were performed to determine the statistical significance of survival differences. Cox proportional hazards model was used for multivariate prognostic analysis. Data were excluded when patients did not continue to follow-up.

## 5. Conclusions

In conclusion, our data demonstrate that ARID1B plays an important role in the growth of *ARID1A*-mutant OCCC. Our most important finding is that low ARID1B expression was associated with shorter progression-free survival. This is the first report demonstrating that low levels of ARID1B protein can serve as a marker of poor outcome in OCCC patients. We also found a significant correlation between the loss of ARID1A immunoreactivity and reduced ARID1B levels, suggesting that ARID1B could be a target for anti-cancer therapy.

## Figures and Tables

**Table 1:** Relationship between ARID1B expression and loss of ARID1A immunoreactivity in OCCC patients.

Intensity of Expression	Patients Number	ARID1A Loss	ARID1A Positive
ARID1B Low	8	4 (50%)	4 (50%)
ARID1B High	45	3 (6%)	42 (94%)

*p* < 0.005 (Fisher’s test).

**Table 2:** Association between ARID1B expression and clinicopathologic factors in OCCC patients.

Factors	Patients	ARID1B Immunostaining		*p*-Value
		Low	High	
FIGO stage				
I, II	40	5	35	0.3547
III, IV	13	3	10	
CA125 U/mL				
<90	27	4	23	0.9538
≥90	26	4	22	
Age (years)				
<54	27	4	23	0.9538
≥54	26	4	22	
Endometriosis				
Without	29	5	24	0.6313
With	24	3	21	
Ki-67				
Low	25	3	22	0.5521
High	28	5	23	
Residual tumor				
<2 cm	41	6	35	0.8627
≥2 cm	12	2	10	

**Table 3:** Univariate analysis of prognostic factors for progression-free survival in OCCC patients.

Factors	Patients	Hazard Ratio	95% CI	*p* Value
FIGO stage				
I, II	40	6.3	2.7–14.7	<0.0001
III, IV	13			
CA125 U/mL				
<90	27	3.5	1.4–9.0	0.0088
≥90	26			
Age (years)				
<54	27	1.7	0.7–4.1	0.2278
≥54	26			
Endometriosis				
Without	29	0.5	0.2–1.3	0.1851
With	24			
Ki-67				
Low	25	1.7	0.7–3.9	0.2323
High	28			
Residual tumor				
<2 cm	41	6.1	2.6–14.2	<0.0001
≥2 cm	12			
ARID1B immunostaining				
Low	8	3.9	1.4–8.9	0.04
High	45			

**Table 4:** Multivariate analysis of prognostic factors for progression-free survival in OCCC patients.

Factors	Patients	Hazard Ratio	95% CI	*p* Value
FIGO stage				
I, II	40	1.6	0.3–8.0	0.5624
III, IV	13			
CA125 U/mL				
<90	27	3.2	1.0–9.8	0.0446
≥90	25			
Residual tumor				
<2 cm	41	3.4	0.7–16.1	0.0692
≥2 cm	12			
ARID1B immunostaining				
Low	8	3.1	1.1–8.7	0.036
High	45			

## Supplementary Materials

Supplementary materials can be found at http://www.mdpi.com/1422-0067/19/6/1710/s1.
