# Supplementary material for: ARID1B as a Potential Therapeutic Target for ARID1A-Mutant Ovarian Clear Cell Carcinoma
Source: Int J Mol Sci. 2018 Jun 8;19(6):1710. doi: 10.3390/ijms19061710 (PMC6032401; doi:10.3390/ijms19061710)
Supplement: Supplementary file 1 [file ijms-19-01710-s001.pdf]

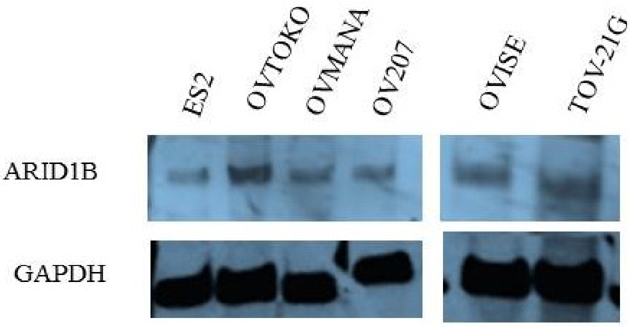

Supplementary Fig. 1. Western blot analysis of ARID1B expression in OCCC cells.

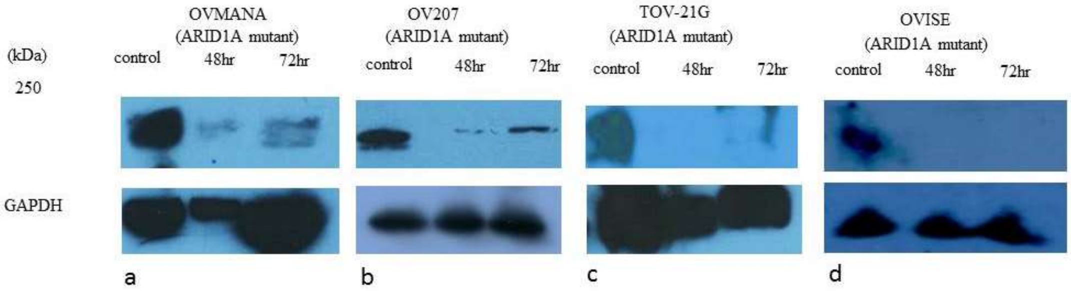

Supplementary Fig. 2. Western blot analysis of ARID1B expression in OCCC cells. ARID1B protein level decreased after siRNA –mediated ARID1B knockdown in ARID1B mutant (a, b, c, d) cells.

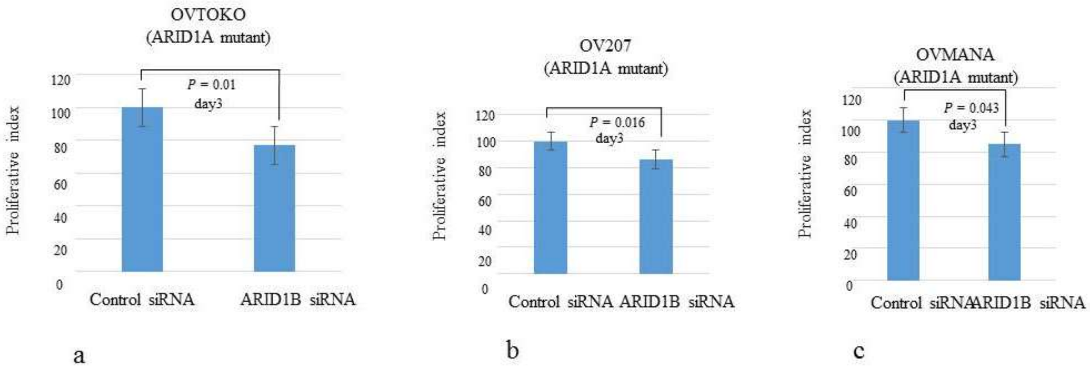

Supplementary Fig. 3. Effect of *ARID1B* knockdown on cell proliferation in OCCC cell lines. *ARID1B* knockdown inhibited proliferation in all *ARID1A*-mutant cell lines (a, b, c)
